# Flexible Carbon Nanotube Films for High Performance Strain Sensors

**DOI:** 10.3390/s140610042

**Published:** 2014-06-06

**Authors:** Olfa Kanoun, Christian Müller, Abderahmane Benchirouf, Abdulkadir Sanli, Trong Nghia Dinh, Ammar Al-Hamry, Lei Bu, Carina Gerlach, Ayda Bouhamed

**Affiliations:** 1 Technische Universität Chemnitz, Chair for Measurement and Sensor Technology, 09107 Chemnitz, Germany; E-Mails: christian.mueller@etit.tu-chemnitz.de (C.M.); benchirouf@ieee.org (A.B.); abdulkadir.sanli@s2012.tu-chemnitz.de (A.S.); nghia.dinh-trong@etit.tu-chemnitz.de (T.N.D.); ammar.al-hamry@etit.tu-chemnitz.de (A.A.-H.); bulei2008@hotmail.de (L.B.); carina.gerlach@etit.tu-chemnitz.de (C.G.); aydabouhamed@gmail.com (A.B.); 2 Higher Engineering School of Sfax (ENIS), University of Sfax, Sfax w.3038, Tunisia

**Keywords:** carbon nanotubes, nanocomposites, piezoresistivity, printed electronics, strain sensors

## Abstract

Compared with traditional conductive fillers, carbon nanotubes (CNTs) have unique advantages, *i.e.*, excellent mechanical properties, high electrical conductivity and thermal stability. Nanocomposites as piezoresistive films provide an interesting approach for the realization of large area strain sensors with high sensitivity and low manufacturing costs. A polymer-based nanocomposite with carbon nanomaterials as conductive filler can be deposited on a flexible substrate of choice and this leads to mechanically flexible layers. Such sensors allow the strain measurement for both integral measurement on a certain surface and local measurement at a certain position depending on the sensor geometry. Strain sensors based on carbon nanostructures can overcome several limitations of conventional strain sensors, e.g., sensitivity, adjustable measurement range and integral measurement on big surfaces. The novel technology allows realizing strain sensors which can be easily integrated even as buried layers in material systems. In this review paper, we discuss the dependence of strain sensitivity on different experimental parameters such as composition of the carbon nanomaterial/polymer layer, type of polymer, fabrication process and processing parameters. The insights about the relationship between film parameters and electromechanical properties can be used to improve the design and fabrication of CNT strain sensors.

## 1. Introduction

Strain sensors have a wide range of applications in engineering, industry and medicine for measuring different quantities, such as stress, torque, pressure and vibration. Despite their excellent features, conventional strain sensors, such as semiconductor and metallic strain gauges, show some limitations considering measurement range, low sensitivity, difficulties to be embedded in material structures, low fatigue life and sensitivity to environment conditions and influencing effects. These limitations have increased the demands for using novel smart materials, e.g., doped silicon [1], nanoparticles [2–4], nanowires [5,6], graphene [7–9] and carbon nanotubes (CNTs) [10–14]. Among these novel sensitive materials, CNTs have become one of the most promising materials since their discovery by Iijima in 1991 [15] and they have attracted a great interest in a wide range of fields because of their exceptional mechanical, electrical, thermal and chemical properties. The excellent properties of CNTs provide interesting opportunities to realize new types of strain gauges, which can overcome some of the performance limitations of conventional commercial metallic strain gauges and allow them to enter completely new application fields.

In this review, we outline flexible CNT films for high performance strain sensor applications. This review is organized as follows: in Section 2 the theoretical background of CNTs is introduced considering structure, electrical, mechanical and piezoresistive properties. Furthermore, in this section fundamentals on percolation theory and the effects of nanofiller alignment on the percolation threshold is also investigated. In Section 3, we emphasize the strain sensor fabrication techniques and strain sensor measurement. Here, we show the most important preparation methods for CNT dispersions and CNT/polymer composites. In Section 4, a comparative investigation of various deposition techniques of CNT films and CNT/polymer composites are briefly discussed. The influence of CNT networks and tunneling effect on the resistivity and the conduction mechanism as well as the piezoresistive characterization of nanocomposite matrix under strain is outlined in Section 5.

Section 6 addresses recent developments in strain sensors based on CNT thin films and CNT/polymer composites by considering the influence of the fabrication parameters, type of CNTs and polymers on the strain sensor behavior and reproducibility. We conclude the review in Section 7 by summarizing the important results, which include limitations of current strain sensors and possible future developments towards flexible strain sensors.

## 2. Theoretical Background

### 2.1. Structure of CNTs

**Figure 1.**
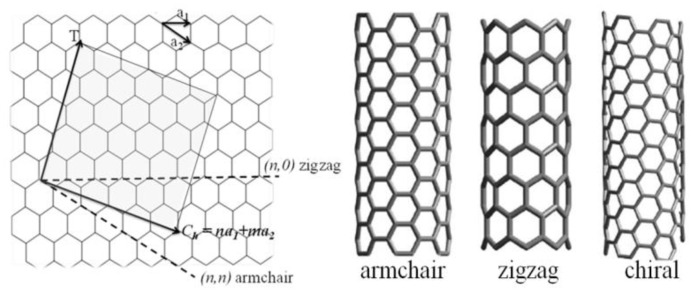
The construction of CNTs from a graphene sheet along the chiral vector *C_h_* [16].

With respect to their physical properties two different forms of CNTs can be distinguished, single walled carbon nanotubes (SWCNTs) and multiwalled carbon nanotubes (MWCNTs). A SWCNT is essentially the rolled-up form of a single graphene sheet. The rolling direction of a graphene sheet is described by the chiral vector *C_h_* = *na*_1_ + *ma*_2_, where *n* and *m* are integers and *a*_1_ and *a*_2_ are the lattice vectors of grapheme, as illustrated in Figure 1 [16]. SWCNTs can be in armchair, zigzag or chiral form depending on the integer values of *m* and *n*. The type of the chirality (*n*, *m*) is decisive for the physical properties of CNTs [16]. For instance, CNTs with “armchair” structure, where *n* = *m* (*n*, *n*), have no band gap and therefore they are always metallic. In the case *m* = 0 (*n*, 0) the structure is called “zigzag” where SWCNT can be either insulating or metallic. The SWCNT is metallic if *n* = 3*q*, where *q* is an integer. The third structure of SWCNT is called “chiral” where *n* > *m* > 0. The SWCNT with chiral structure can be metallic if *n* − *m* = 3*q*.

MWCNTs consist of multiple rolled-up graphene sheets in two different structures called *russian doll model*, where graphene sheets are rolled up in concentric cylinders and the *parchment model*, where one graphene sheet is rolled like a scroll of parchment [17]. MWCNTs are always metallic and their conductivity is quite complex due to the various coaxially-arranged SWCNTs [18]. The electron transport in the MWCNTs is found to be similar to that of SWCNTs because most of the current passing through the tube is limited to the outermost layer [19,20].

### 2.2. Mechanical Properties of CNTs

CNTs have a high stiffness and axial stress due to the covalent sp^2^ hybridized bonding between carbon atoms [21]. Scientific measurements using *in situ* atomic force microscopy (AFM) and transmission electron microscopy (TEM) have been conducted to estimate the Young's modulus and it is in the range of 270 GPa to 950 GPa [22,23]. In comparison with conventional materials, CNTs show also high tensile strength of up to 63 GPa [24], due to the extra energy absorption required for the hollow structures of carbon nanotubes. Mechanical properties of CNTs have a strong dependence on nanotube structural details such as electronic band structure, e.g., size dependent Young's modulus for small size SWCNT [25], tensile behavior dependence on helicity, diameter and defects. The effects of mechanical deformation *i.e.*, kinking, sliding and compression were studied both experimentally and theoretically by quantum mechanical simulations [26]. Mechanical tests inside a high resolution TEM were carried out by measuring the change of resistance of individual MWCNT when mechanically slided and kinked [27]. Tips from atomic force microscopy (AFM) were used to deflect suspended individual SWCNT and caused a decrease of two orders of magnitude of conductivity due to the formation of local sp^3^ bonds between tube and tip [28]. When applying reversible deformations and compressive strains (bending) on individual SWCNTs with an AFM tip alterations of the band gap and the conductivity were observed [29]. A strain gauge was theoretically predicted by the tight-binding approach and for SWCNTs with diameter larger than 1 nm it was found to be chirality dependent [30].

### 2.3. Electromechanical and Piezoresitive Properties of CNT Networks

The electromechanical and piezoresistive properties of randomly distributed CNT networks and CNT/polymer composites were studied experimentally and theoretically [31–34]. CNT/polymer composites have a percolation behavior whereby the interconnections between CNTs network form conductive paths. The role of the polymer in the network is considered by the tunneling barrier *i.e.*, the gap between neighboring tubes and tunneling barrier height. A box of three-dimensional statistical resistors network model using a tunneling effect between neighboring nanotubes represents the CNT network. The overall network resistance consists of the resistance of the CNT filler and the tunneling resistance. In addition, capacitance is considered where a three-dimensional capacitance network, *i.e.*, gap capacitance between neighboring tubes and capacitance between the electrodes by material, was also simulated and allowed study in the frequency domain [32]. In numerical simulation a tunneling distance between neighboring CNTs is considered and Kirchhoff's current law and Ohm's law are used to obtain the electrical current and conductivity. The piezoresistivity of the CNT network comes from three main parts; the conductive paths formed by CNTs, tunneling between neighboring CNTs and CNT piezoresistivity. Conductive paths and tunneling effects were found to play the major role in the piezoresistivity of the CNT network [31]. Further explanations with respect to CNT resistor network and tunnelling mechanism are given in Section 5.

### 2.4. Percolation in CNT Networks and the Influence of Process Parameters on the Electrical Properties

**Figure 2.**
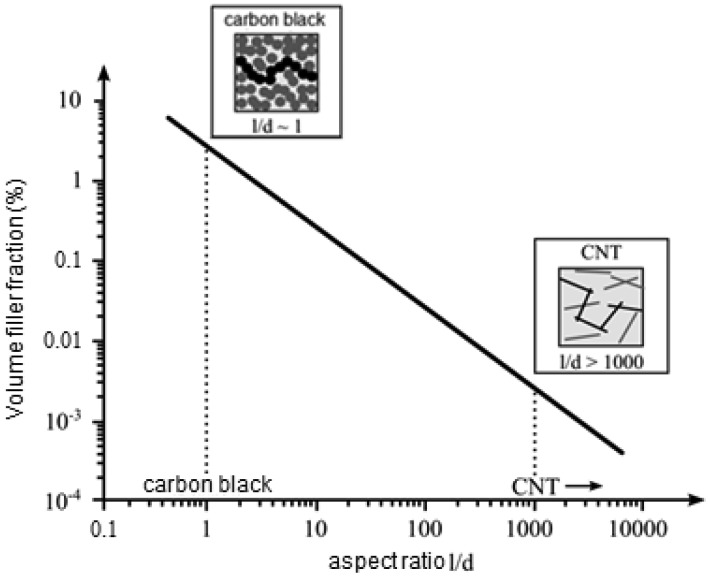
Volume filler fraction as a function of the aspect ratio *l*/*d* (length/diameter) exemplarily shown for carbon black and CNTs.

A high strain measurement sensitivity can be reached at the percolation threshold [10,31,35]. The percolation threshold depends on different factors such as CNT aspect ratio, CNT type, shell quality, dispersion degree and the functionalization of the CNTs. Generally, CNT networks with functionalized CNTs have higher percolation thresholds than non-functionalized CNTs. Numerous studies have shown that the percolation threshold and conductivity depend strongly on the polymer type, fabrication parameters, aspect ratio of CNTs (Figure 2), disentanglement of CNT agglomerates, uniform spatial distribution of individual CNTs and degree of alignment [22,36]. Therefore, percolation thresholds ranging from less than 0.5 wt % to over 10.0 wt % of CNTs loading have been observed experimentally [37–39]. It is expected that the addition of CNTs to a polymer significantly enhances the conductivity of the composite. In general, the electrical conductivity of heterogeneous systems above the percolation threshold can be described by a scaling law [40]:

(1) $$\sigma =\sigma_{0}{\left(\theta -\theta_{c}\right)}^{t}$$

where *θ* is weight fraction of the conducting filler, *θ_c_* corresponds to the percolation threshold, and *t* refers to the critical exponent. According to the percolation theory, the critical exponent depends only on the dimensionality of the system, *i.e.*, *t* = 1.6 for two and *t* = 2 for three dimensions.

Wang *et al.* [41] dispersed two different kinds of MWCNTs (non-functionalized and COOH functionalized MWCNTs) with the same dimensions in silicon rubber, and they found that functionalized MWCNTs samples had four times higher resistance than non-functionalized MWCNTs.

**Figure 3.**
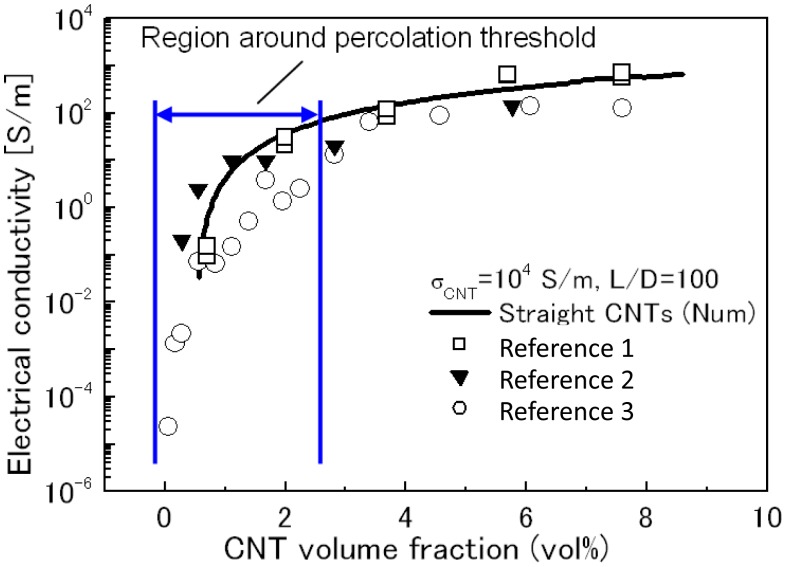
Electrical conductivity as a function of CNT volume fraction [42].

In the case of non-functionalized CNTs with an aspect ratio of 1000, Bauhofer and Kovacs [36] showed that the percolation threshold might be obtainable at 0.1 wt% for nearly any optimized CNT/polymer composite. Above a concentration of 2 wt%, the conductivity reaches the saturation level [42,43] (Figure 3).

**Figure 4.**
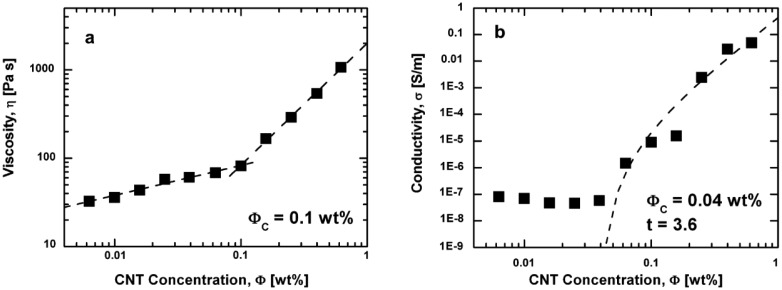
Viscosity and conductivity of MWCNT/epoxy composites. (**a**) viscosity depending on the filler concentration. (**b**) conductivity depending on the filler concentration [36].

An important aspect, especially for the fabrication, is the step gradient of viscosity above the percolation threshold. A relationship between viscosity and conductivity was reported by Bauhofer *et al.* [36] (Figure 4).

**Figure 5.**
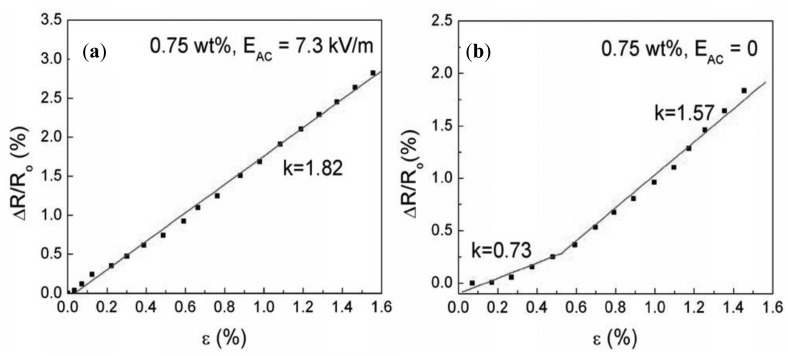
Piezoresistive characterization of MWCNTs/Polysulfone films. (**a**) 0.75% MWCNTs, E_AC_ = 7.3 kV/m (aligned). (**b**) 0.75% MWCNTs, E_AC_ = 0 kV/m [45].

Alignment of CNTs in the composite can help to achieve low percolation thresholds. Grossiord *et al.* [44] reported a high conductivity of 1000 S/m for aligned MWCNTs (2 wt%) in polystyrene. Avilés *et al.* [45] aligned MWCNTs in polysulfone by applying alternating electrical fields E_AC_ of 6 kV/m and 7.3 kV/m. At low CNT concentrations (0.1 wt%–0.5 wt%), the conductivity of the aligned composite can be up to five orders of magnitude higher than that of randomly distributed CNTs. However, the conductivity is similar at higher CNT concentrations (0.75 wt%). Aligned MWCNTs showed a linear behavior of conductivity over the whole measurement range (Figure 5a), whereby the randomly distributed CNTs showed two different regions (Figure 5b) [45].

## 3. Preparation of CNT Dispersions and CNT/Polymer Composites

Agglomerates in nanocomposites deteriorate their electrical and mechanical properties and decrease their homogeneity, therefore realization of dispersion is a decisive step in the fabrication of CNT composites, especially for the realization of reproducible CNT-based structures with predictable properties. In general, CNTs have a mixture of various chiralities, diameters, lengths and defects. CNTs have a high aspect ratio and large surface areas, therefore they tend to assemble into bundles due to the Van der Waals attractive force between tubes [34]. CNTs are also hydrophobic and they have poor solubility in aqueous solutions and organic solvents [46].

Several processing methods are possible for fabricating CNT films and CNT/polymer composite films based on aqueous dispersions or polymer composites. The use of different polymers, such as thermoplastic, thermoset and elastomer matrices has been reported [47–49]. They mainly include methods to individually and homogeneously disperse CNTs within a solvent or polymer matrix [36,50–52] such as solution mixing, melt mixing, bulk mixing and *in situ* polymerization or combinations of them. In this section we address a selection of relevant processing techniques for both aqueous and polymer based dispersions.

### 3.1. Non Covalent Functionalization of CNTs

Covalent bonding of functional groups to the sidewalls and ends of CNTs can be achieved by chemical functionalization. This process can be performed with reactive molecules, such as fluorine, hydrogen, radicals, or aromatic cycles. Defect functionalization is another approach for covalent functionalization of CNTs. Defect sites are open ends, holes or deviations from the hexagonal graphene framework. The defect sites on CNTs, which can be created by strong oxidants such as HNO_3_ or H_2_SO_4_, are stabilized with carboxyl or hydroxyl groups, allowing further chemical modifications and improved solubility of the CNTs in hydrophilic solvents. However, chemical functionalization and the corresponding preparation steps induce many defects, which are detrimental to the mechanical properties of the CNTs. Therefore, non-covalent or physical functionalization methods have been developed to disperse CNTs. Besides the wrapping with polymers, various non-ionic surfactants, such as polyoxyethylene octylphenylether (Triton X-100) [53]; anionic surfactants, such as sodium dodecylsulfate (SDS) [54], sodium dodecyl benzene sulfonate (SDBS) [55,56]; and cationic surfactants, such as dodecyl trimethyl ammonium bromide (DTAB) [57] have been employed for physical functionalization. In [55], it was proved than when using SDBS as surfactant higher percentages of single tubes could be obtained than when using SDS and Triton X-100 [55]. The good dispersion quality with SDBS was also confirmed by optical spectroscopy [56]. Generally, ionic surfactants are preferable for dispersing CNTs in aqueous solutions and nonionic surfactants are suitable for dispersing CNTs in organic solvents [58]. The efficiency of the dispersion depends strongly on the properties and concentrations of solvent, CNTs and polymers.

### 3.2. Solution Mixing

The most common method to disperse CNTs and to fabricate CNT/polymer composites is solution processing. In general, the fabrication method includes the dispersion of CNTs in a solvent medium by mechanical mixing, magnetic stirring or sonication, mixing the CNT dispersion with the polymer solution and evaporation of the solvent.

**Figure 6.**
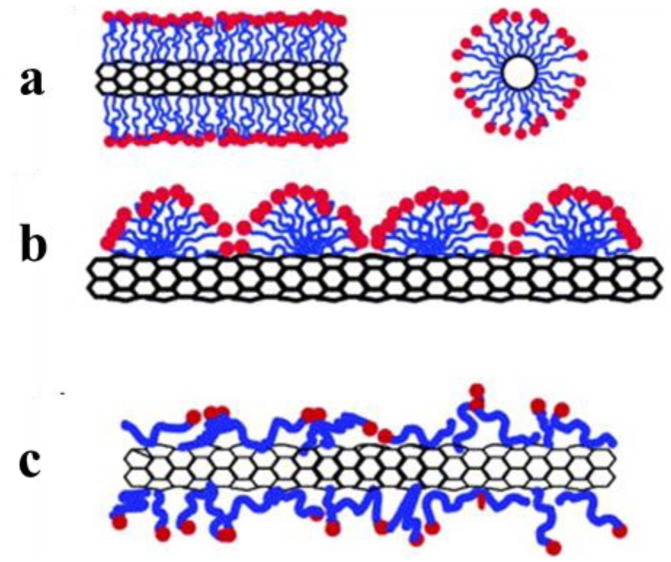
Mechanism by which surfactants help to disperse SWNT. (**a**) SWNT encapsulated in a cylindrical surfactant micelle (both cross section and side-view). (**b**) Hemimicellar adsorption of surfactant molecules on a SWNT. (**c**) random adsorption of surfactant molecules on a SWNT [59].

Surfactants are very important for dispersion of carbon nanotubes. The interaction between CNTs and dispersion differs significantly depending on the chemical composition and concentration of the surfactant (Figure 6). For the dispersion of CNTs it is assumed that the minimum surfactant concentration below the critical micelle concentration is necessary in order to realize uniform and stable dispersions [59].

**Figure 7.**
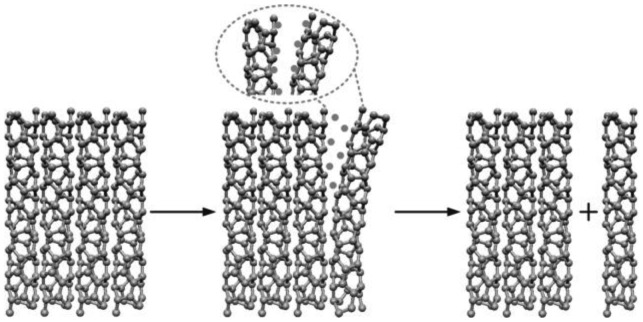
Schematic view of the unbundling of CNTs by surfactant interaction and sonication processes.

The mechanical treatment of the CNT solution is very important for the dispersion quality (Figure 7). The duration of mixing processes and the concentration of the surfactant influence the dispersion quality and the quantity of remaining agglomerates. Better unbundling is generally achieved by a higher surfactant concentration and longer processing times within a certain range. However, too intensive mechanical processing may lead to changes of the CNTs and introduce more defects. A high surfactant concentration leads to more residuals after drying processes, which influence the electrical properties of composite films. Therefore a compromise between surfactant concentration and processing time is required to achieve good unbundling. Furthermore, centrifugation processes are used to remove remaining CNT bundles.

### 3.3. In-Situ Polymerization

**Figure 8.**
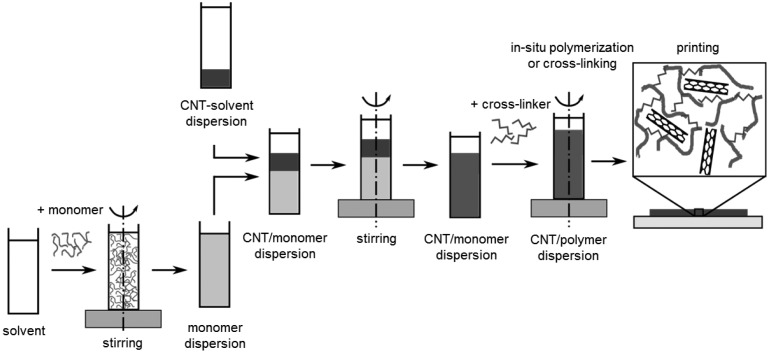
Fabrication of CNT/polymer composites using a combination of *in-situ* polymerization and solution mixing.

*In-situ* polymerization of vinyl monomers in the presence of CNTs has been intensively studied for the fabrication of functional composites. This technique produces polymer-grafted CNTs mixed with free polymer chains [60]. Due to the small size of the monomeric molecules, the homogeneity of the composite is much higher than that obtained by mixing CNTs and polymer chains. In this sense, the method is suitable for the preparation of composites with enhanced mechanical properties due to strong interfacial bonds. Initially, *in-situ* radical polymerization has been successfully used for the synthesis of PMMA composites [61]. A combination of both *in-situ* polymerization and solution mixing is a promising approach for fabrication of polydimethylsiloxane (PDMS) composites [38]. A generalized approach of this fabrication process is summarized in Figure 8.

### 3.4. Melt Processing for Polymer Composites

Thermoplastic polymers, such as polypropylene (PP) [62] or polystyrene (PS) [63], soften when heating above their melting point and can be utilized as matrices for CNT-based polymer composites. By blending the polymer melt with CNTs high shear forces are applied leading to a better homogeneity of the composite. Depending on the final morphology and shape of the composites, the samples can be further processed by several techniques, for example, extrusion or spinning [58]. Compared with composites prepared by solution mixing, the degree of dispersion of CNTs achieved by melt processing is lower and the fabrication is limited to small amounts of CNTs. Melt processing has the advantage that it can be used for insoluble polymers which cannot be processed with solution mixing.

## 4. Deposition of CNTs and CNT/Polymer Composites

Deposition is required to transfer the dispersion or composite material from its initial liquid state onto a desired substrate. The quality of the obtained films depends on the rheological properties of the composite, the substrate material, the surface pretreatment and the properties of the deposition technique itself. In particular, the deposition technique influences the adhesion, the force transfer and the force distribution of the film. Therefore, an appropriate deposition process is crucial for achieving high quality films and good sensing properties. Various deposition techniques, such as drop casting, layer by layer deposition, inkjet printing, spin coating, spray coating, vacuum filtration and Meyer rod coating can be used to deposit CNT films. In this section those methods are briefly discussed. It should be noted that the development of nanocomposites is a growing field and therefore the number of deposition methods will be increasing in future.

### 4.1. Drop Casting

Drop casting is a simple and cheap technique to fabricate CNT films. Normally the prepared CNT dispersion is cast into a masked structure. After the evaporation of the solvent, the CNT film is formed and adheres to the substrate surface due to the Van der Waals forces [64]. Changing the CNT concentration, the volume of the CNT dispersion and the number of deposited layers can control the thickness of the fabricated CNT film. By using drop casting, large area CNT films can be realized. The thickness of the generated CNT films typically spans several hundred nanometers to several micrometers [64]. The reached homogeneity of the CNT films with drop casting technique is limited. Due to the differential evaporation rates of the solution with dispersed particles, the so called “coffee ring effect” is often observed.

### 4.2. Layer-by-Layer Deposition

The layer-by-layer (LBL) self-assembly technique uses attraction forces such as electrostatic hydrogen bonding between the deposited species to deposit thin films onto the desired substrate. The thin films are fabricated by an alternative immersion of the substrate into two oppositely charged electrolytes (anionic and cationic) with a washing step in between. Thin films of a few nanometers can be reached. A higher film thickness could be realized by repeating these coating steps. This simple deposition technique is very powerful as it gives the ability to assemble complex structures on the nano-scale range at low cost [65–70].

### 4.3. Inkjet Printing

One of the potential advantages of solution-processed CNTs is the possibility of implementing inkjet printing to enable high-throughput large-area fabrication. In recent years, many researchers have investigated inkjet printing as a new deposition method to fabricate CNT films [71–73]. Different printing technologies to deposit CNTs on various substrates were subject to investigation, such as aerosol printing [74–76], screen printing [77] and contact printing [78]. Results show that using printing techniques has potential for fabricating low cost CNT devices and sensors [71]. By inkjet printing macroscale structures can be directly patterned on substrates without the use of masks, photolithography and etching processes. In addition, various substrates can be used in the printing process, for example paper, polymer film, glass, wafer, and ceramic. Several investigations have shown promising results demonstrating the use of inkjet printing to fabricate CNT based flexible electronics [76,79–83]. Both organic solvent-based carbon nanotube inks [81–84] and water-based carbon nanotube inks with the use of dispersants have been developed [85–87]. Kordas *et al.* [71] reported large-area patterning of CNTs on paper and on polymers using a commercially available inkjet printer. In [73] SWCNTs, MWCNTs and functionalized CNTs were used to produce inks by mixing with the conductive polymer poly(3,4-ethylenedioxythiophene) (PEDOT). A piezoelectric inkjet printer was used to generate patterns on polymer films. Various printing parameters, such as voltage, frequency, drop spacing, substrate temperature and nozzle temperature were studied. Sheet resistances of the printed patterns were measured and compared with each other. The results indicated that functionalized CNTs are the best candidate to prepare the conductive CNT ink. By using functionalized CNTs (CNT-PEG) together with PEDOT-PSS [50:50] the lowest sheet resistance (225 Ω/sq) was achieved [73]. There are still some obstacles to the use of this deposition method, for example, nozzle clogging due to the presence of CNT agglomerates in the ink, relative slow speed and the microscopic inhomogeneity caused by the coffee ring effect that still has to be investigated.

### 4.4. Spin Coating

Spin coating is a technique for CNT film deposition and it is preferable for coating thin CNT films in the range of a few nanometers to hundreds of nanometers [88–90]. In contrast to other methods the film thickness can be easily controlled by the speed and coating time, various substrates can be used and the coating process can be performed at room temperature.

Kim *et al.* [90] used spin-coated dispersions of SWCNTs dispersed on glass substrates and achieved transparent and surfactant free SWCNT films, which have a root-mean-square roughness of 2.0 nm measured by AFM and a sheet resistance of 128 Ω/sq.

### 4.5. Spray Coating

Spray coating is similar to spin coating. It can be also used to deposit CNT films on various substrates up to large sizes. This deposition technique has been adopted to fabricate functional CNT devices [90–93]. However, spray-coated films have a higher roughness than those deposited by using spin coating [90].

### 4.6. Other Deposition Methods

Vacuum filtration and Meyer rod coating were reported in different studies [94–97]. Vacuum filtration [94–97] has the advantage that the thickness of the filtrated CNT films can be easily controlled by the concentration and the volume of the CNT in the dispersion. The drawbacks of this technique are the limited film size of the filter and the necessity to transfer the films to more suitable substrates [95]. Meyer rod coating is another widely used deposition process for the fabrication of CNT films, because of its simple use for industrial mass products [96]. To apply Meyer rod coating defined rheological properties of the CNT dispersion are required.

## 5. Resistance of CNT Films and Strain Measurements

In order to understand the electrical conduction mechanism of the CNT film, the changes of the whole CNT network under strain have to be considered. In fact, not all CNTs within a CNT film contribute to the electrical conduction. The conductance of the CNT film is induced by the CNT network, which forms conducting paths between the electrodes. If each CNT in the film is assumed as a straight stick, the two-dimensional stick percolation theory [97], which is comprehensively explained in Section 2.4, helps to reveal the electrical conduction mechanism of composites consisting of CNTs and an insulating matrix. When the conducting filler content is gradually increased, the composite undergoes an insulator-to-conductor transition. The critical filler content is referred as the percolation threshold where the electrical conductivity of the composite sharply increases several orders of magnitude due to the formation of conducting paths. Consequently, at the percolation threshold the sensitivity of the strain sensor is high. Below the percolation transition range, such conducting paths do not exist and the electrical properties are dominated by the matrix material. At filler amounts above the percolation transition range, multiple conduction paths exist and the electrical conductivity of the composite achieves a saturation level. When the composite is under strain, the configuration, position and orientation of the CNTs in the network change, that leads to significant modification of the conducting paths. In addition, the geometry and the area of the CNT film change also under applied strain. All these factors interplaying together lead to the film resistance changes under strain.

A CNT film can be seen as a network formed by a large number of randomly arranged individual CNTs and small CNT bundles. Two types of resistances determine the resistance of a CNT film. The first type is the intrinsic resistance *R_tube_* of the CNT itself. Typical values for *R_tube_* of MWCNTs are in the range of 0.2 kΩ·s/µm to 0.4 kΩ·s/µm. The second type is the intertube resistance *R_junction_*. Then the total resistance of the CNT film can be calculated as:

(2) $$R=R_{\text{tube}}+R_{\text{junction}}$$

The *R_junction_* part can be further divided into the contact resistance *R_C_* for CNTs in physical contact, and tunneling resistance *R_T_* for CNTs separated by a small gap. The tunneling resistance *R_T_* can be estimated by [98]:

(3) $$R_{T}=\frac{h^{2}d}{Ae^{2}\sqrt{2m\lambda }}e^{\frac{4\pi d}{h}\sqrt{2m\lambda }}$$

where *d* is the distance between CNT, *e* is the quantum of electricity, *h* is the Planck constant, *m* is the electron mass, *λ* is the barrier height of energy and *A* is the cross sectional area of the tunnel. From Equation (1) can be seen that *R_T_* increases nonlinearly, resulting in a nonlinear piezoresistivity.

The working mechanism in piezoresistive CNT strain sensors is mainly attributed to: (i) variation of conductive CNT networks or loss of contacts among CNTs, affecting *R_C_*; (ii) distance change between neighboring CNTs, promoting *R_T_* and (iii) deformation of CNTs themselves, varying *R_tube_*. However, due to poor stress transfer from the polymer matrix to the CNTs the contribution of *R_tube_* to the piezoresistivity of the CNT strain sensor is expected to be very small. Hu *et al.* [99] used a combined 3D resistor network and fiber reorientation model to explain the working mechanism in piezoresistive CNT networks. They found that under low strains the resistance of composites with CNT concentrations (<1%) close to the percolation theshold is dominated by the tunneling effect instead of breakup of electrical contacts. At high CNT concentrations the piezoresitivitity was found to be almost linear. CNT networks within a composite or film also dominate the resistance-temperature behavior. The temperature response of a CNT network can be described based on a modified Lutinger liquid model and Fermi liquid theory by:

(4) $$R(T)=R_{\text{tube}}T^{-\alpha }+R_{\text{junction}}T$$

where *α* is a constant value and *T* is the temperature [100].

When a CNT film is under strain, the change in the film resistance is the result of changes in both *R_tube_* and *R_junction_*. The change in *R_tube_* under strain is due to the variation of the band-gap of individual tubes. This effect depends therefore on the chirality of individual tubes and has an exponential behavior with the strain [101]. Under stress, *R_junction_* changes with the varying inter-tube distances. Thereby, both contact and tunneling resistance change. This effect depends on the length and concentration of CNTs. Especially in the region of the percolation, the changes of *R_junction_* are more dominant in comparison to changes of *R_tube_*.

Despite significant investigations [102,103], fundamental understanding of the piezoresistive behavior in CNT/polymer composites still needs to be investigated. The total sensitivity of a piezoresistive CNT film can be quantified using the gauge factor *K*, which is defined as the relative change in electrical resistance with respect to the strain:

(5) $$K=\frac{l}{\text{d}l}\frac{\text{d}R}{R}$$

where d*R*/*R* is the relative change in the resistance, generated by the applied strain ε = d*l*/*l*. The *K* factor for CNT films can go up to 80 [30]. For classical metallic conductors (copper, nickel), this *K* factor is typically around 2. This high value of *K* for CNT films can be explained by two factors: the change of geometry of the sensor and the change in the percolation network of the system. Aforementioned points are very important for increasing the resolution of strain measurement, even by using low cost electronic components and AD-converters.

## 6. Strain Sensors Based on Carbon Nanotube Thin Films and CNT/Polymer Composites

A systematical investigation of the influence of process parameters, such as the sonication time and surfactant concentration on the corresponding CNT dispersions is described in this section. These factors are decisive for the quality and properties of CNT films prepared from CNT dispersions.

### 6.1. CNT Film Strain Sensors

**Figure 9.**
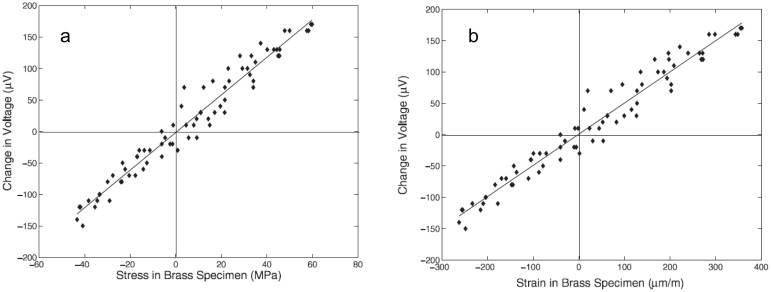
BP-CNT films subjected to compression and tension. (**a**) change in voltage as function of stress. (**b**) change in voltage as function of strain [96].

One approach to realize CNTs films is to fabricate a buckypaper (BP). The first studies that considered BP-CNTs for strain sensing were performed by Dharap *et al.* [96] and Li *et al.* [103]. They used SWCNTs dispersed in *N,N*-dimethylformamide (DMF), and the mixture was filtered through a Teflon membrane. A BP-CNT film with isotropic properties was achieved, due to the random orientation of SWCNTs. Dharap *et al.* observed a linear change in voltage across the film subjected to a tensile or compressive force as it is shown in Figure 9 [96]. Li *et al.* observed a linear relationship between the shift of the bands in the Raman spectra, *i.e.*, G-Band and D-Band, and the tension force [103].

**Figure 10.**
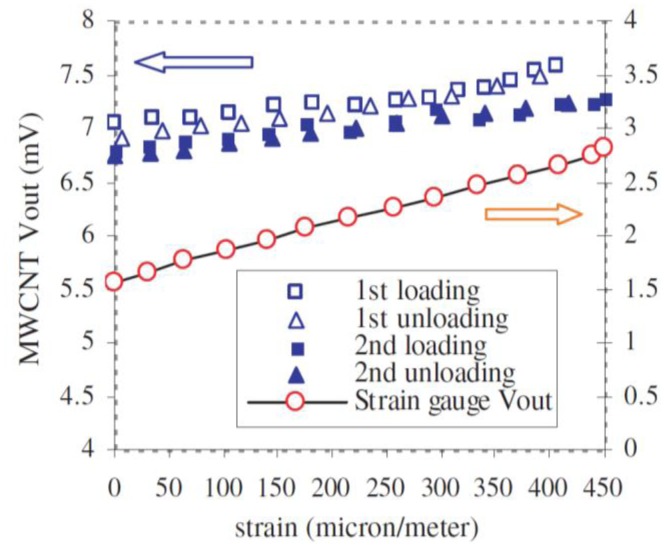
Voltage output of MWCNT film and foil strain gauge under applied strain [105].

SWCNT films have relative high reproducibility restrictions due to the influence of chirality, impurities and electrical properties on the strain sensing behavior [30,104]. MWCNTs were proposed to overcome these limitations [105–107]. Li *et al.* [105] and Vemuru *et al.* [106] showed in 2008 the possibility of using MWCNT films for strain sensing. Both used a BP film from the Nanolab company. The MWCNTs were suspended with NanoSperse AQ surfactant in water. A membrane was utilized to filter the suspension. The freestanding film was peeled off from the membrane after the drying process was complete. Li *et al.* [105] focused mainly on the response of MWCNT films to static loading and on resistance change with temperature, which showed an independency behavior, although a small drop of 0.1 Ω within a temperature range from 273 K to 363 K was observed. In addition, they performed dynamic sinusoidal loading tests and the results showed a good response for MWCNT films at high frequencies with up to one order of magnitude higher than the foil strain gauge [105] (Figure 10).

Vemuru *et al.* [106] used the four-point probe technique to measure the change in voltage of MWCNTs-BP attached to a brass specimen in a tension test. A linear change in voltage across the film when subjected to tension could be measured. The MWCNT film showed high recovery properties, which means low hysteresis, and stable electromechanical properties for loading and unloading states, but low gauge factors of around 0.35.

Miao *et al.* [64] prepared SWCNT films in the absence of surfactants based on the Kaempgens method [108] and used them for in-plane strain sensing. The piezoresistive behavior of the pure CNT films was investigated under applied longitudinal (in-plane) tensile strain. A linear relationship between the change in the resistance and the applied strain was observed. The gauge factor of the film was found to be 2.6 and 2.4, respectively, at compressive and tensile strain. Moreover, a low hysteresis behavior was observed.

**Figure 11.**
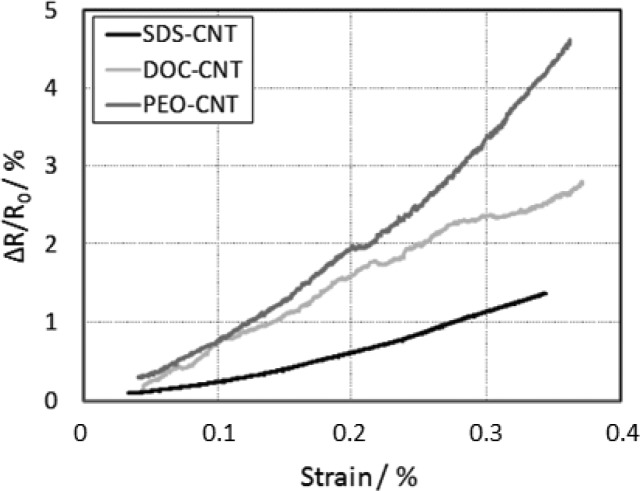
Relative change of resistance of MWCNT strain gauges dispersed in different surfactants.

Dinh *et al.* [109] compared the piezoresistive properties of films based on MWCNTs dispersed in different surfactants such as SDS and deoxycholic acid (DOC) as well as MWCNT/polymer composite with a polyethylene oxide (PEO) matrix. Films were fabricated by solution drop casting on defined rectangular shapes on tensile specimens. The substrates were loaded in the extensometer for stretching measurements and the related change in the film resistance was recorded simultaneously. All the fabricated films showed a linear dependence of the sensitivity under the strain. The strain-resistance characteristics show a quadratic behavior, with a strain sensitivity of 4, 7.5 and 12.5 for SDS, DOC and PEO, respectively (Figure 11). However, among them the films based on MWCNT/SDS give the most stable mechanical behavior. The observed fluctuation is mainly due to inhomogeneity of the films.

**Figure 12.**
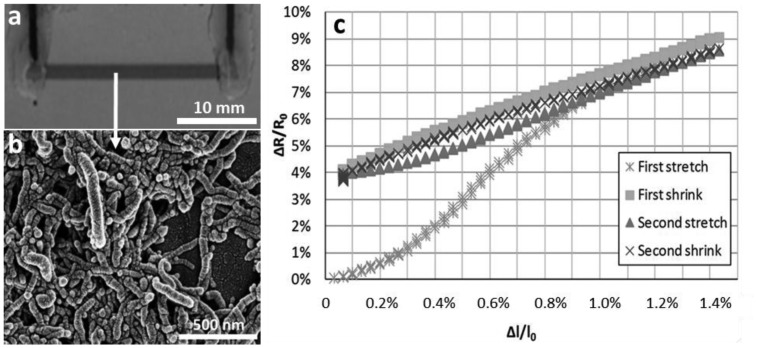
MWCNT films deposited by inkjet printing. (**a**) Optical microscopy image of a CNT film with a size of 2 mm × 30 mm. (**b**) corresponding SEM image of the CNT film. (**c**) change of resistance *vs.* strain at first and second cycle in tenile test redrawn from [110].

**Figure 13.**
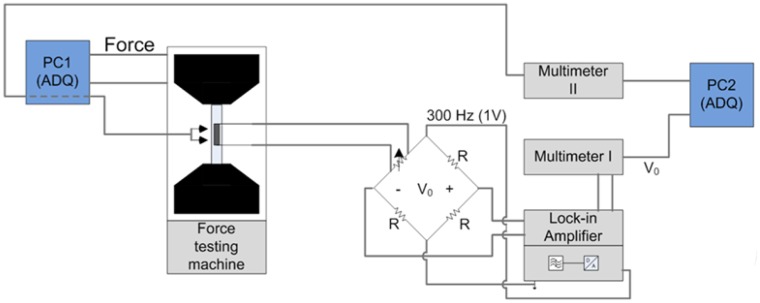
Strain measurement setup [110].

Dinh *et al.* [110] used inkjet printing to deposit the MWCNT/SDS dispersions on flexible PET substrates (Figure 12a,b). The relationship between the applied strain and the change in the resistance was analyzed by loading the samples in tensile test. The results show a linear response already at the second measurement cycle (Figure 12c). In order to monitor the resistance changes in CNT films, a Wheatstone bridge circuit was coupled with a lock-in amplifier (Figure 13).

**Figure 14.**
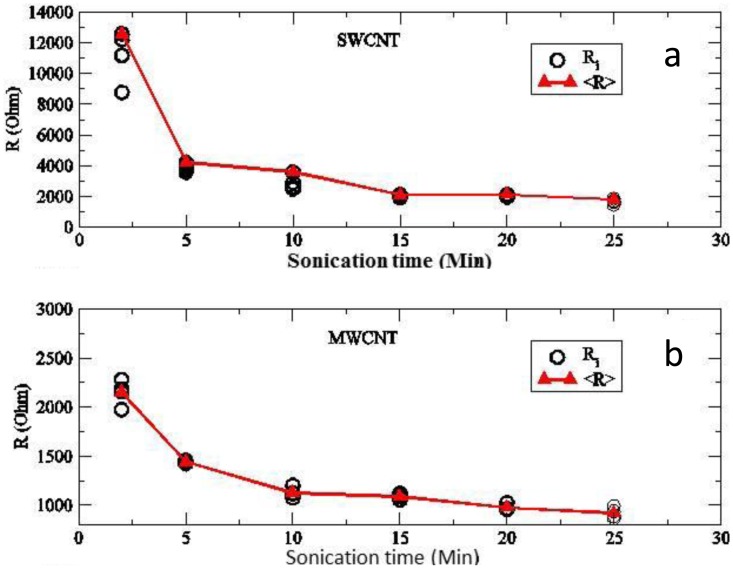
Resistance *vs.* sonication time. (**a**) for 0.1 wt% SWCNT dispersed in SDS. (**b**) for 1 wt% MWCNT dispersed in SDS [64].

The effect of processing parameters such as sonication time and concentration of surfactant have been studied as well in [70,111]. Bu *et al.* [64] showed the influence of these process parameters on the resistivity of CNT films. It was demonstrated that the sonication time strongly influences the quality of the CNT dispersion. Uniform CNT films and reproducible resistances for SWCNT (Figure 14a) and MWCNT (Figure 14b) were achieved by an appropriate sonication time.

**Figure 15.**
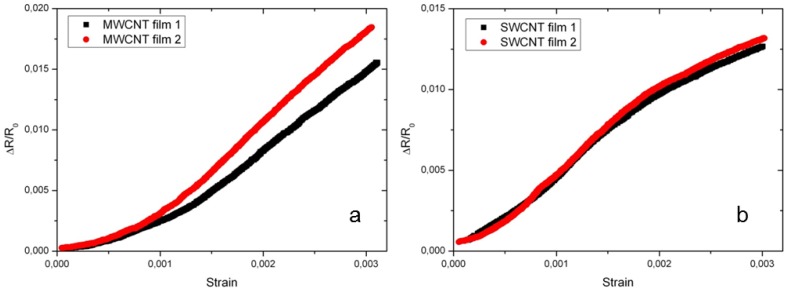
The relative change in film resistance *versus* the applied strain for drop casted CNT films. (**a**) MWCNT (IoLiTec Ionic Liquids Technologies GmbH) films prepared by using MWCNT dispersions with 1 wt% MWCNTs, 0.5 wt% SDS and 15-min sonication time. (**b**) SWCNT (Array) films prepared using the SWCNT dispersion with 0.1 wt% SWCNTs, 0.5 wt% SDS and 15-min sonication time [64].

For MWCNT films, two regions were observed. The sensitivity of MWCNT films is found to be about 2.5 for the first region corresponding to strains smaller than 0.1%. For the second strain region from 0.1% to 0.3%, a linear trend between the applied strain and relative change in resistance was identified, with a gauge factor of 5 as shown in Figure 15a. These two different behaviors can be explained as follows. In the low strain region, the sensitivity is attributed to the change in the contact resistance between the CNTs. Whereas, at higher strain, an accumulation of contact resistance change of the CNTs and the deformation in the electronic structure *i.e.*, the bandgap of the individual tubes occurred, which results in a higher sensitivity [54,70,112]. At strains smaller than 0.2% SWCNT films showed a similar behavior as MWCNTs films (Figure 15b). At strains above 0.2%, the behavior could be explained as poor load transfer due to the slippages of the SWCNTs.

**Figure 16.**
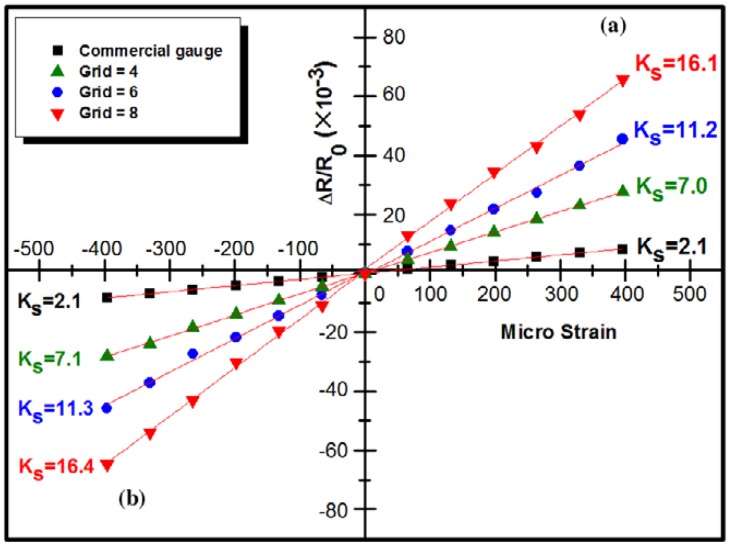
Resistance–strain curves for spray-coated SWCNT film strain gauges under (**a**) tensile. (**b**) compressive load [93].

Unlike the BP-CNT films fabricated in [96,102], Lee *et al.* [93] used spray coating to deposit a SWCNT dispersion on grids bonded to a flexible polyimide base. In an attempt to control the gauge sensitivity of the spray-coated SWCNT film, design parameters such as a strain-sensitive CNT grid and the film thickness were experimentally varied to quantify the changes in the performance of the spray-coated SWCNT strain gauges. Lee *et al.* [93] showed that the proposed SWCNT film gauges have a linear relationship between resistance change and applied strain. Different gauge factors could be obtained from 7.0 to 16.4 for four different micro-grid configurations (Figure 16).

Yin *et al.* [113] investigated the piezoresistive behavior of aligned SWCNT forests and found along the two key directions of the film an almost linear relationship between resistance change and strain. The longitudinal and transversal gauge factors were measured to 3.75 and 0.67, respectively.

### 6.2. CNT/Polymer Composite Strain Sensor

CNT/polymer composites have improved mechanical properties compared to pure polymers. Commonly CNTs embedded in a polymer have higher sensitivity than CNT films without polymer. Dinh *et al.* reported about a sensitivity of 12.5 by using DOC as tenside [109]. On the other hand a maximum sensitivity of 22.4 was reported in epoxy [93]. In general, CNT/polymer based strain sensors can be applied to several measurement ranges due to consideration of different kinds of polymers. Depending on the polymer also strains of lower than 0.25% typically applied to metal DMS can be detected. However, the unbundling process of CNTs in a polymer is a challenging task. Additionally, the attachment between CNT and polymer and between composite and the measurement object need further development.

Different possibilities were investigated for dispersions of CNT/polymer composite strain sensors, such as sonication, stirring and calendering. For thermoplastic polymers melt processing, stirring and hot pressing are preferred [36]. For elastomers and thermosets *in-situ* polymerization is often used, which leads to a good cohesion between the CNTs and polymer. For deposition, very often mould casting and screen printing is favoured. In some investigations LBL technology is used [113–115]. For the same CNT concentration, CNT/polymer composites in elastomer matrix have a higher conductivity than thermoplastic and thermoset based composites. For instance, Mechrez *et al.* [116] reported that the conductivity could reach up to 1000 S/m for polyacrylate with 10 wt% CNTs. Chen *et al.* [117] found a conductivity of 280 S/m for randomly distributed MWCNTs (1.3 wt%) in PDMS . In contrast, in thermoset and thermoplastic matrices the conductivity ranges from 0.1 S/m to 50 S/m with the same CNT concentration [118–121].

The sensitivity of nanocomposites can be predicted by measuring the viscosity of the dispersion. This fact can be helpful for the development of strain gauge with high sensitivity. In order to achieve a high sensitivity, a high conductivity in the saturation area is not useful (see Figure 3). Based on the study of Ramasubramaniam *et al.* [115] for SWCNT/PPE composites the highest sensitivity can be achieved in the conductivity range from 0.1 S/m to 10 S/m.

Sensitivity data obtained from SWCNT/polymer and MWCNT/polymer composites are summarized in Table 1. For an easy comparison of the data it was assumed that 1 vol % SWCNTs is equivalent to 1 wt.% and 1 vol.% MWCNTs is equivalent to 2 wt.% [36].

Generally, a lower concentration leads to lower conductivity and therefore a higher sensitivity. The best gauge factor in the studied literature (see Table 1) reported by Yin *et al.* [113] is 22.4. For epoxy as polymer, they used *in-situ* polymerization and applied planetary mixing. It was found that alignment of CNTs leads to improved linearity of the strain dependent resistance change. Whereas, randomly distributed CNTs have two ranges with different sensitivity in their strain/resistance characteristic [112,113].

Unlike metallic strain gauges, which have a low resistance, the high resistance of CNT/polymer composite is a challenge for signal processing. Due to the presence of tunneling effects within the composite, the conductivity of the nanocomposite depends strongly on the temperature. Furthermore the temperature noise increases with increasing resistance. Therefore, comprehensive investigations have to be made between temperature cross-sensitivity, temperature noise and the sensitivity.

In order to explain the experimental results theoretical investigations have been performed. Thereby effects like destruction and formation of conductive paths which take place when a strain is applied were considered. However, the dominant effect in sensitivity is not predictable yet. Especially the increasing resistance in elastomers by extension and compression shows a discrepancy with the simulation results [62].

**Figure 17.**
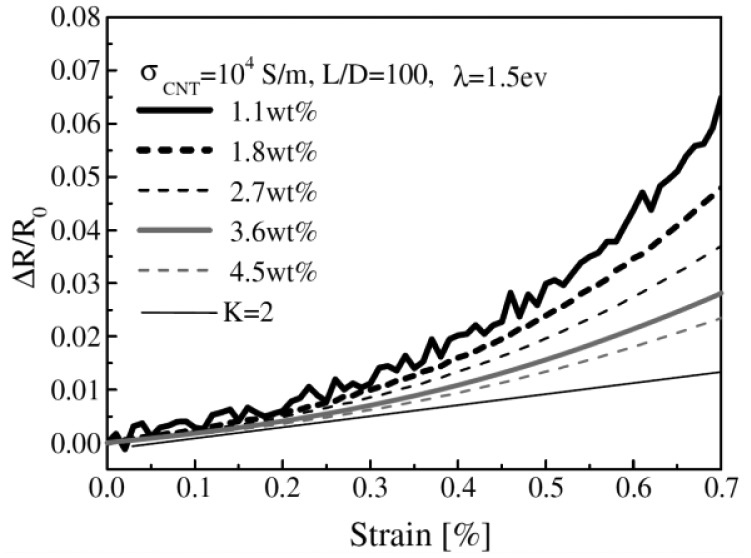
Numerical calculation of resistance change depending on strain for different MWCNT concentrations [31].

At low CNT concentrations in the nanocomposite large conductivity fluctuations may occur due to the strong influence of destruction and formation of conductive paths [120]. This behavior has been experimentally and theoretically confirmed by Hu *et al.* [31] (Figure 17).

Recently, a lot of progress has been achieved in increasing the strain sensitivity and understanding the working mechanism, which is of high importance for practical applications. However, up to now only a few studies about the stability and reproducibility of CNT strain sensors have been reported. There are some examples related to structural health monitoring [11,123–125]. For instance, Yamada *et al.* [11] used SWCNT on PDMS to measure human motion by assembling the sensors on bandages or on clothes. Furthermore, these sensors were used for real time monitoring of biological functions of the human body such as breathing and phonation.

Another interesting option for CNT-based films are wireless strain sensors. Conventional strain sensors can only measure the strains directly on the structural surface, therefore cables need to be applied, and this makes it difficult for some applications, e.g., vibrational applications. Hence, there is a need to develop wireless strain sensors which can work in harsh environments and detect strains wirelessly. For this purpose CNT films can be easily patterned. Loh *et al.* [126] used SWCNT/PVA LBL deposition to pattern a coil antenna onto a flexible PET substrate and measured wirelessly the impedance response of the films under strain.

## 7. Conclusions and Outlook

In this contribution, CNT-based films for high performance strain sensors on flexible substrates have been reviewed. Besides the outstanding physical properties of CNTs, limitations of CNTs such as the tendency to aggregation, hydrophobicity and poor solubility in aqueous and organic solvents influence the properties of CNT-based nanocomposites. In order to achieve an efficient preparation of CNT dispersions and CNT/polymer composites we focused firstly on the effects of various surfactant types and deposition techniques on CNTs' physical properties. It was found that the efficiency of CNT dispersion strongly depends on the properties of solvents, CNT and polymer types. For instance, using SDBS as surfactant leads to better dispersion in comparison with SDS and Triton X-100 surfactants.

The parameters that influence the resistance properties of CNT films can be described based on the two dimensional stick percolation theory which helps to understand the electrical conduction mechanism of CNT-based nanocomposite matrices. The percolation threshold and conductivity depend strongly on the polymer type, fabrication parameters, aspect ratio of CNTs, disentanglement of CNT agglomerates, uniform spatial distribution of individual CNTs and degree of alignment. Furthermore, a strong influence of the fabrication parameters on the CNT films strain sensor performance and reproducibility were shown.

Experimentally, it was found that the strain sensor sensitivity is greatest at the percolation threshold of CNT/polymer based nanocomposites that depends on many factors, e.g., aspect ratio, CNT type, shell quality, dispersion degree and functionalization of the CNTs which in turn defines the sensitivity of CNT/polymer composite strain sensors. For this purpose, an overview has been given for determining the effects of different type of filler, polymer and fabrication process on the strain sensitivity.

By comparing the sensitivity of SWCNTs and MWCNTs, it can be concluded that SWCNTs add no advantage to CNT film strain sensors although they show intrinsic semiconducting and metallic behavior in the CNT network. From experimental and theoretical findings, it is difficult to predict which effect is dominating when strain is applied and conductivity fluctuation occurs at low CNT concentration due to destruction and formation of conducting paths.

Consequently, controlling and understanding the influence of the CNT film parameters on the sensor properties is crucial for strain sensor applications. Although numerous studies have been conducted, the CNT-based strain sensors still have to be investigated more in parallel to the development of nanotechnology until they will achieve the desired properties for commercial applications.

## Figures and Tables

**Table 1.** Experimental parameters for CNT/polymer film and corresponding strain sensor parameters. Acronyms: PMMA (polymethyl methacrylate), PVA (polyvinyl acetate), PSS (poly(sodium 4-styrene-sulfonate)), PSF (Polysulfon), PEO (polyethylene oxide).

**Filler (Diameter; Length)**	**Polymer**	**Fabrication Method**	**Conductivity [S/m]**	**Gauge Factor (Amount of CNT)**	**Ref.**

SWCNT	PMMA	buckypaper filled with PMMA	-----	5.3 (0.5 wt.%)-1 (10 wt.%)	[10]

SWCNT	PVA, PSS	solution mixing; layer by layer, thin film	-----	0.208	[118]

SWCNT	PVA, PSS	solution mixing; layer by layer, thin film	-----	1.805	[119]

MWCNT (60–100 nm; 0.5–500 µm)	PMMA	bulk mixing then melt processing (film: 0.127 mm)	-----	15.32 (1 wt.%)	[120]
				4.59 (3 wt.%)	
				4.26 (5 wt.%)	
				3.27 (6 wt.%)	
				1.9 (8 wt.%)	
				1.44 (10 wt.%)	

MWCNT (10–20 nm; Several µm)	epoxy	*in-situ* polymerization (three roll mill); mould casting	1 × 10^−4^	0.75 (0.1 wt.%)	[121]

MWCNT (10–20 nm; Several µm)	epoxy	*in-situ* polymerization (three roll); mould casting	2 × 10^−4^	4.5 (0.1 wt.%)	[122]
			1.32 × 10^−2^	3.5 (0.3 wt.%)	

MWCNT (10 nm; 5–15 µm) small and curved shapes	epoxy	*in-situ* polymerization (planetary mixer); mould casting	7.07 × 10^−4^ (5 wt.%)	4.9 (5 wt.%)	[113]
			1.92 × 10^−3^ (7 wt.%)	4.5 (7 wt.%)	
			5.33 × 10^−3^ (10 wt.%)	5.8 (10 wt.%)	
			4.49 × 10^−2^ (15 wt.%)	4.4 (15 wt.%)	

MWCNT (40–90 nm; 5–10 µm) straight shapes	epoxy	*in-situ* polymerization (planetary mixer); mould casting	10.4 (5 wt.%)	22.4 (1 wt.%)	
			65.8 (7 wt.%)	7.6 (4 wt.%)	
			95.2 (10 wt.%)	6.2 (5 wt.%)	
			125.1 (15 wt.%)	4.8 (7 wt.%)	
				3.2 (10 wt.%)

MWCNT (40 nm; 4–6 µm)	epoxy	*in-situ* polymerization (planetary mixer); mould casting	0.02 (1 wt.%)	22.4 (1 wt.%)	[31]
			1 (2 wt.%)	12 (2 wt.%)	
			10 (5 wt.%)	6 (5 wt.%)	

MWCNT (40–90 nm; 10–30 µm)	epoxy	*in-situ* polymerization (planetary mixer)	3.3 × 10^−2^ (1 wt.%)	22.4 (1 wt.%)	[123]
			1.13 (2 wt.%)	11.6 (2 wt.%)	
			10.4 (5 wt.%)	6.2 (5 wt.%)	

MWCNT (4–13 nm; 1–4 µm)	PSF	solution mixing then mould casting in AC electrical field (aligned)	-----	2.68 (0.5 wt.%)	[45]
				1.82 (0.75 wt.%)	

MWCNT (4–13 nm; 1–4 µm)	PSF	solution mixing then mould casting; (randomly distributed)	-----	0.73 (0.75 wt.%, <0.5% strain)	
				1.57 (0.75 wt.%, >0.5% strain)

MWCNT	PEO	solution mixing; mould casting	-----	3.7 (1.12 wt.%, <0.85% strain)	[112]
				1.6 (2.9 wt.%, <2% strain)	
				50 (2.9 wt.%, >2% strain)	
